# History of a Case in Which Very Uncommon Worms Were Discharged from the Stomach; with Observations Thereon

**Published:** 1800

**Authors:** Samuel Crumpe

**Affiliations:** M.R.I.A.


XXIII. *Hiſtory of a Caſe in which very uncommon Worms were diſcharged from the Stomach; with Obſervations thereon.* *By* Samuel Crumpe, *M. D. M. R. I. A.* *From the Tranſactions of the Royal Iriſh Academy.* Vol. VI.

THE lady whoſe caſe I am about to relate had been for many years ſubject to inflammatory affections of the lungs, which generally terminated in a copious and long continued expectoration I was called to her aſſiſtance for the firſt time on the 2d of November 1788; ſhe was then about twenty-ſix years of age and had been about two years married. She appeared to me to labour under the ſymptoms which characterize the advanced ſtages of phthiſis pulmonalis, and which need not here be enumerated; and to theſe, according to her own account, ſhe had been more or leſs liable every winter and ſpring for ſome years back. As her ſweats were very profuſe, and ſhe complained much of troubleſome cough, and want of reſt, the principal remedies preſcribed were thoſe calculated to reſtrain the preſſing ſymptoms; and conſiſted chiefly of the weak vitriolic acid, and thebaic tincture. By theſe ſhe found herſelf relieved until the 22d of December, when ſhe was ſeized with an evident peripneumony, attended by conſiderable pain under the ſternum, which required, and yielded to, copious blood-letting and bliſters. It terminated as uſual in an abundant expectoration, and ſhe recovered from it but ſlowly. About the latter end of February, however, ſhe began to gain ſtrength and ſoon after was to all appearance perfectly recovered.

On the 14h of July, 1789, I was again called to her, and found her again labouring under the ſymptoms of pneumonic inflammation; from which ſhe was by the uſual mode of treatment freed in four or five days.

*August* 4th.—I was again called on. She has been troubled for four or five days paſt with a vomiting of blood, which generally recurs twice or thrice in the twenty-four hours; and is preceded by a ſenſe of weight and oppreſſion about the præcordia, which are relieved by the vomiting. The quantity thrown up is various at different times; ſometimes a tea-cup full, ſometimes not two table-ſpoon fulls, and generally in clots. Complaints alſo of want of ſleep, profuſe night ſweats, tendency to cough, which is prevented by great ſoreneſs in her cheſt, want of appetite, and ſometimes difficulty of breathing; pulſe natural in point of frequency, but very full; belly regular. She was bled to about eight ounces; blood remarkably ſizy.

*August* 6.—Symptoms as before, excepting the pulſe, which is very natural. She was ordered an infuſion of the bark, with the vitriolic acid, and a mucilaginous opiate.

*August* 8.—Has continued to take the infuſion regularly; vomiting of blood ſtill continues; ſleep rather better; cough eaſy; ſweats continue. Let the infuſion be continued.

*August* 12.—Took the infusion regularly. Vomiting of blood continues; sweats as before, cough easy, and expectoration free.

*August* 13.—Has taken the infusion regularly. Vomiting of blood has ceased; but she is troubled with retchings and vomiting every morning, preceded by sweats, and a gnawing sensation in the stomach. In vomiting this morning says she threw up three small worms, which have not been preserved. Cough has disappeared; sweats decreasing, except before the morning vomiting; sleeps pretty well. Continue.

*August* 24.—The vomiting has recurred at intervals without any worms having been thrown up, 'till this morning, when a considerable number of small ones, and of uncommon shape, were discharged. Sweats have almost entirely ceased, and she is in other respects well. Continue the infusion.

*September* 3.—Was seized this morning with a return of vomiting, when she threw up a considerable number of worms similar to the last, and one of a much larger size and different shape, mixed with bloody and corrupted matter; the small ones were so numerous they could not be reckoned, the entire matter thrown up being full of them. No cough, dyſpnœa, or other diſtreſſing ſymptom.

R. Stanni pulverati drachmam, Salis Martis granum; fiant pulveres tales octo, & ſumat unum quater de die.—Repetatur Infuſum Corticis.

*September* 5.—Has taken the whole of the powders; ſlight returns of vomiting; no appearance of worms; ſhe was ordered and took a vomit of ipecacuanha-wine, which operated well, but neither worms or bloody matter were diſcharged. The infuſion was ordered to be continued.

*September* 26.—Sweats have diſappeared; cough is but ſlight; the vomiting pretty frequently recurs, but is not preceded by any unuſual ſenſation, nor is it more frequent than in her former pregnancy. No appearance of blood or worms; ſtrength in general much improved.

She was ordered to omit all medicines, was ſafely delivered of her child at the uſual time, and is now in perfect health; though in general delicate, and ſubject to violent colds.

*Obſervations.*

*Obfervations.*

The worms thrown up are delineated of the natural fize and appearance in fig. 8, and 9. (Plate II.) Fig. 8, reprefents the large one, confifting of a head and twelve joints; the three firft joints are furnifhed each with a pair of legs, which all the others want. Fig. 9, reprefents the fmall worm, of which fuch numbers were difcharged; and fig. 10, the belly of the fame, viewed through a good common magnifying-glafs. It confifts of a head and ten joints, and has three double rows of legs; one double row on each fide, and one double row of fhorter ones down the middle of the belly. It has befides three legs projecting in the form of a tail from the laft joint.

All thofe worms fhewed figns of animation when difcharged, efpecially on being expofed to the heat of the fun, but foon died. They appear to me to be the larvæ of fome infect, but of what particular fpecies, I am not naturalift minute enough to determine. The large one appears very fimilar to the larva of the common beetle. We have many inftances related by by various authors, of different ſpecies of worms diſcharged from the inteſtine canal: but of the different deſcriptions I have read, or ſpecimens I have ſeen preſerved in anatomical collections, none have ſtruck me as in any degree ſimilar to thoſe diſcharged by the patient whoſe caſe has been juſt related. It is probable, as has before been mentioned, that the worms diſcharged were the larvæ of ſome inſect which does not uſually depoſit its eggs in any part of the human frame; but which having been accidentally depoſited in, or conveyed into the body, were hatched, and acquired the ſize and form we have delineated.

That flies of various kinds depoſit their eggs in living animal bodies, and that theſe eggs are by the heat of the animal hatched and transformed into maggots, is evident from the inſtance of many ſo produced in the rectum of horſes, and backs of black cattle. The human body being in general better covered, and better defended from the attempts of ſuch inſects, does not exhibit ſo many inſtances of this nature. Still, however, it is liable to their attacks. Many caſes have been publiſhed of various inſects being hatched, and producing excruciating excruciating pain in the antrum maxillare, and other cavities leading to the nose. Other worms, evidently the larvæ of external insects, have been discovered in the intestines: And in the Medical Commentaries for the year 1787 there is a curious case of some existing under the skin.

A young lad of about twelve years of age was afflicted with excruciating pains in his limbs, to such a degree as to render life miserable. The pains were deemed rheumatic, and the usual remedies for that disorder applied without success. At length some worms worked their way out of different parts of his body, particularly the knees, breast, and forehead; and he was immediately after considerably relieved. The worms are described as near an inch long, all in joints on the back, and with hard scales on them. The attending physician ascribes their origin to some flies having pierced the skin and lodged their ova in the punctures. Several other boys in the same part of the country were that season affected in a similar manner.

Many instances have been given by medical authors of inflammatory and other affections of the lungs occasioned by worms, some of which proved proved fatal; ſome being relieved, or totally removed by the diſcharge of theſe animals. Morgagni in the 2d book of his incomparable work *De Cauſis & Sedibus Morborum*, gives us the caſe of a patient, who laboured under every ſymptom of pleuriſy, which terminated with his ſpitting up a quantity of blood with a round worm; after which he immediately got well. He in the ſame place quotes a work publiſhed by Ignatius Pedratti, on the pleuriſy from worms.

We have other inſtances of worms diſcharged from the lungs by coughing, in Schenkius *Obſerv. de Pulmonibus*, lib. 2, in Lieutaud *Hiſt. anatom. med.* in Percival's Eſſays, and in the works of ſeveral other medical writers.

It may perhaps be imagined that the pulmonic affections under which our patient laboured might have been owing to the worms ſhe diſcharged, which perhaps worked their way downwards from the lungs into the ſtomach; this, however, does not appear probable; her having been ſubject to them for a ſeries of years, their being frequently excited ſuddenly by the application of cold, and their terminating without the diſcharge of any ſuch animals, animals, are circumſtances which militate ſtrongly againſt the idea.

There can, however, I believe, be little doubt that the complaints of the ſtomach with which ſhe was ſeized, and the vomiting of blood, were occaſioned by their preſence; and that they formed for themſelves a nidus in the coats of the ſtomach, appears pretty evident from the purulent and bloody matter which accompanied the diſcharge of the laſt portion of them.

None of the ſymptoms attending the vomiting of blood were ſuch as indicated the preſence of worms in the ſtomach and inteſtines; nor when their preſence was aſcertained did the vermifuge medicines given appear to accelerate the cure; ſtill, however, the caſe may be inſtructive, as proving that the hæmatemeſis or vomiting of blood ſometimes ariſes from this ſource, and therefore that the phyſician ſhould keep ſuch a cauſe of the diſorder in view whenever it proves obſtinate or dangerous; and the hiſtory is curious, as affording one certain fact reſpecting the nidification of inſects in the internal parts of the human frame.

*Limerick,*

*December* 1, 1794.

